# Supplementary material for: Individual and community-level benefits of PrEP in western Kenya and South Africa: Implications for population prioritization of PrEP provision
Source: PLoS One. 2020 Dec 31;15(12):e0244761. doi: 10.1371/journal.pone.0244761 (PMC7775042; doi:10.1371/journal.pone.0244761)
Supplement: Attachment — Submitted filename: Response to Reviewers.docx [file pone.0244761.s003.docx]

**Response to Reviewers' comments:**

5. Review Comments to the Author

**Reviewer #1:** This paper presents an agent-based network model of HIV transmission among heterosexuals in Western Kenya and South Africa. The study compares the impact of introducing PrEP to either men or women, with variable scenarios based on age group provision and exogenous processes (i.e., increased ART and male circumcision) across varying time periods. The authors found that PrEP provision to women had the greatest immediate impact and was also the most efficient allocation of resources.

Overall, the study is well-designed and relies on a network model which has been used in other studies. The model complexity is appropriate for this type of study. I appreciate that the authors present a number of counterfactual scenarios which could be helpful for policy makers. The manuscript is timely, as resources for public health may be limited (i.e., due to coronavirus) so policy makes may need to be strategic about allocating resources for other priorities. However, the overall presentation of the methods and results should be improved before the manuscript is ready for publication.

Major points:

1. The methods section does not provide sufficient information for a model of this complexity. The authors point to some references, but it was not immediately obvious which references were most relevant. I suggest the authors rework the methods section to provide greater detail about the overall structure of the model and some key parameters which are relevant to the research question. If word count is a problem, it is common for models of this complexity to have a technical appendix. One of the references (13) was helpful to answer some of my questions, but this was published in 2012 so it is not clear what, if anything, has changed about the model and code since then. It may also be helpful to remove the references to your group’s other studies (refs 11 and 12) using this model and just point the readers to the reference that is clearly describing the model design. If the other references are relevant to piece together changes to the model since 2012, then I think it would be most appropriate to compile a single document (i.e., technical appendix) which clearly describes the current model. Reference 10 is too general to provide much relevant information for the present study.

**Response:** Methods and associated citations have been substantially re-written to provide additional clarity on what EMOD is (a codebase that supports modeling of many different infectious diseases), the transmission modalities in the EMOD-HIV model, and the procedures and parameters used to calibrate the model to the two settings in our analysis. We have added citations for each of these components of the methods, including working links where readers can find detailed definitions and allowable ranges for all model parameters. We have added a reference to a mathematical paper (Klein et a., *IEEE Conference on Decision and Control*) that describes in technical detail the implementation of the sexual network in the EMOD HIV model. The two papers by Selinger et al. describe the fitting of the model to the South African HIV epidemic and baseline projections for incidence and prevalence in the absence of PrEP. The paper by Bershteyn et al. in *Infect. Dis. Modeling* similarly describes the fitting of the western Kenya model. Supplementary Table 3 of that paper lists all calibrated model parameters and their values and ranges.

Pages 4 to 5 lines 84 to 115 in Revised manuscript with track changes

2. The sexual network characteristics seems to be the most important information that was left out, since this is the underlying mechanism driving the model results. Summary information about concurrency and assortativity should be provided so that readers can better understand differences between men and women (i.e., degree, concurrency and anything else that is relevant) and how this is generating the model results. I don’t think it is necessary to have all parameters summarized in the main text of the manuscript, as this would obviously be too much information. I know that some of this information is listed in the excel files provided, which I appreciate, but these are summarizing the calibration so it is unclear to me what the final numbers were and whether there is a single calibrated parameter for each that is consistent across counterfactual scenarios. It would be really helpful to summarize specific numbers as well as words in the manuscript.

**Response:** We have added additional detail regarding the sexual network and edited the overall Methods section for length while retaining key details and references. Crucially, we now reference a detailed technical description and validation of the sexual network, which is configured using a feed-forward algorithm that ensures a stable age pattern of relationship formation even with changes in underlying demographics (see Klein et a., *IEEE Conference on Decision and Control*). In addition, Table S4 has been edited to indicate key parameters used and the median value for each of the key parameters, many of which pertain to sexual behavior such as concurrent sexual partners.

Pages 4 to 5 lines 84 to 115 in Revised manuscript with track changes

3. The results section was challenging to follow. There are many counterfactual scenarios presented and it was difficult to keep track of each. Additionally, the authors present the results qualitatively, rather than quantitatively, for some sections. I suggest the authors add outcome measures throughout; ideally, some kind of a point estimate, such as a median, as well as an interval, such as a 95% simulation interval.

**Response:** We have re-written the description of the baseline scenarios to clarify this point. There are two baselines (one for the status quo scenario and the other for the fast track scenario) and four intervention scenarios representing different PrEP strategies. Table 1 describes the two baseline scenarios, which represent either a pessimistic (status quo) or optimistic (fast-track) level of ART and male circumcision scale-up (Table 1). The status quo baseline assumes continuation of current rates of uptake of HIV services (other than PrEP), while the fast-track baseline assumed achievement of programmatic targets. Specifically, in the fast-track baseline, ART coverage is assumed to reach UNAIDS 95-95-95 targets for 2030. Circumcision is assumed to reach South Africa 2017-2021 National Strategic Plan targets and 100,000 circumcisions yearly from 2022-2036 (to maintain 80% coverage) and in western Kenya, it achieves 95% coverage among men ages 15-24 years by 2020. The intervention scenarios simulate the effect of PrEP offered to populations defined by age (15-24 and 15-49) and gender (male and female) against the backdrop of each of the two baseline scenarios.

We have avoided reporting of absolute impact metrics from the model due to the unresolved uncertainty of PrEP coverage and adherence. Indeed, under current fiscal challenges, it may be some time until PrEP is widely available. Instead, our article seeks to provide insight into how to prioritize different sub-populations for PrEP given the direct as well as the indirect benefits of PrEP. We feel this is best conveyed in relative rather than absolute terms, i.e., the direct-to-indirect impact ratio.

Page 8 to 10 from line 143 in Revised manuscript with track changes

4. There are no tables presenting results of any kind, which I was surprised to see. This would be helpful to summarize the results, but also could serve as a way to guide readers through the various counterfactual comparisons that are presented and how the outcomes increase/decrease across varying conditions.

**Response:** Please see our response to item 3 above.

Minor issues:

Abstract:

1. The Results section presents no quantitative results of any kind, so there is little difference in what is written in the Results section vs. Conclusions. Also consider re-writing this section so that it is clearer what the counterfactual contrasts are which are producing the results. (I know abstracts are short and appreciate that this is challenging).

**Response:** We have revised the Results section with more specific, quantitative outcomes as suggested by the Reviewer.

Page 2 to 3 lines 37 to 46 in Revised manuscript with track changes

2. Line 40: I’m a little confused by the last sentence in this section, as my interpretation of the results is that total infections averted varied based on time (prioritizing immediate results) and assumptions about fast-track vs status quo.

**Response:** We have omitted this confusing statement.

Page 3 line 46 in Revised manuscript with track changes

3. The Conclusions (e.g., first sentence, line 43) are clearer than what is written in the Results section, although both are saying essentially the same thing.

**Response:** We are happy to hear that the Conclusions section is clearer and have revised the Results section to improve its clarity as well.

Introduction:

4. Line 50: The 54% number is ambiguous as written. Does this refer to 54% of new infections? Overall prevalence?

**Response:** We have revised the manuscript to clarify that this is 54% of the global population living with HIV.

Page 3 line 56 in Revised manuscript with track changes

5. Line 52: I certainly don't disagree that incidence is unacceptably high, however this implies that there is some threshold for which ongoing transmission would be acceptable. Perhaps remove the word “unacceptably”?

**Response:** We have now removed the word unacceptably from this sentence. Thank you for the suggestion.

6. Lines 63-64: Some references for the sentence “However, sexual networks play…” would be helpful since not all readers will be familiar with network science and models. Two suggestions (though there are others that would be appropriate): Rothenberg "How a net works", STD, 2001; and Liljeros "Sexual networks: implications for the transmission of sexually transmitted infections" Microbes and Infection, 2003.

**Response:** We have added these references. Thank you for the suggestions.

7. Line 66-67: Again, a reference here would be helpful. The two above may be relevant. Also, Morris has some relevant references (e.g., "Concurrent partnerships and the spread of HIV," AIDS, 1997).

**Response:** We have added these references. Thank you for the suggestions.

Methods:

8. Line 76: I’m glad to see a link to the github repository. However, is there a specific repository for the code used for the present study?

**Response:** We have provided a link for the repository where the EMOD code can be found. We have also revised the Methods section and added appropriate references to clarify that the EMOD code base supports modeling of multiple infectious diseases including HIV, tuberculosis, and malaria. The compiled code supports all of these diseases, with much of the code shared across diseases, so that there is not a specific repository solely for the HIV model. However, there is online documentation and a parameter dictionary specifically for the HIV model, and there are publications specific to the structure of the HIV model, and we have provided the link and associated references.

Page 4 lines 84 to 89 in Revised manuscript with track changes

9. Line 77: The idmod.org link is broken. I found this page <https://idmod.org/docs/emod/hiv/hiv-model-overview.html> which I think is relevant and helpful, but doesn’t provide sufficient information for how this specific study was implemented.

**Response:** Thank you for your diligence in checking the hyperlink. The link has been updated in the manuscript.

Page 5 lines 88 in Revised manuscript with track changes

10. Line 78: What is the age structure of the model? When do individuals enter the model? Do they leave at age 49? Or is that just the cutoff for PrEP eligibility?

**Response:** We have revised the Methods to clarify that the population demographics are input to reflect the actual demographics (fertility, mortality, age/sex distribution) of the respective settings. We have furthermore provided references to more detailed descriptions of our model configuration for each of the two settings used in the analysis. We performed only a minor update to the model calibration to incorporate the latest survey data, as described in the revised Methods section, and Table 4 + Supplementary Table 5 now list all parameters that have been adjusted with the updated data.

Page 5 lines 95 to 99 in Revised manuscript with track changes

11. Line 79: How is transmission operationalized? Condoms are not mentioned in the manuscript at all. How does ART impact transmission probabilities?

**Response:** We have added additional detail describing how transmission is operationalized at the level of the individual coital act. Condom usage parameters are provided in Table 4 and Supplementary Table 5

Page 5 lines 93 to 102 in Revised manuscript with track changes

12. PrEP Parameterization: Were any other aspects of the PrEP clinical protocol modeled? What was the frequency of HIV screening? Was this the same as individuals not using PrEP? Does knowledge of HIV infection (via screening and diagnosis) change behavior (e.g., condom use) in the model? What about initiation of PrEP or ART?

**Response:** We have added clarification that PrEP was implemented in EMOD as a reduction risk of HIV acquisition per coital act applicable to those who take up and adhere to PrEP, and time-limited to the period that these individuals are retained on PrEP. No other aspects of PrEP clinical protocol were considered in this study.

Page 6 lines 117 to 123 in Revised manuscript with track changes

We also added in the Discussion section as a limitation of our study and a possible area for further research.

Page 13 lines 283 to 285 in Revised manuscript with track changes

13. Line 91: It seems PrEP effectiveness was the same for all individuals using PrEP. Was there any heterogeneity in how this was parameterized (e.g., operationalized via varying adherence)? Is adherence known to vary between men and women (or by age?) in this population? This seems like an important limitation that should be either addressed in the model or discussed.

**Response:** PrEP effectiveness is the same for all individuals. We note that PrEP efficacy is higher among highly-adherent individuals, and low among less-adherent individuals. We therefore consider 75% efficacy to be a weighted average of transmission rate in coital acts among those adherent to PrEP as compared to coital acts among those not prescribed or not adherent to PrEP.

Page 6 lines 120 to 123 in Revised manuscript with track changes

14. Line 92: Why is long-acting PrEP mentioned? Is this just to say that the model was limited to oral PrEP?

**Response:** We have now removed the text referring to long acting PrEP.

15. Line 106: Does 45% coverage refer to 45% of all women in scenarios targeting PrEP to women? Or is this 45% among the women of a specific age group? (Same questions for men).

**Response:** It is 45% of the women/men in the age group being targeted for PrEP. We have added “in the corresponding age/sex category” on page 7 lines 137 to 138 in Revised manuscript with track changes to clarify this point.

16. Line 130: The time ranges are listed as 10 or 20 year horizons, but the results refer to 4 year, 8 year (possibly other) timelines. My suspicion is that the authors ran models for 10 or 20 years, but looked at results across other ranges, which is fine technically, but is confusing for how the results are presented. It would be helpful to provide clarity.

**Response:** We thank the reviewer for pointing out this potential point of confusion. We have changed the 10- and 20- year horizons to only 20-year horizon (the total time duration analysed 2016 to 2036). The results section presents what happens within this 20-year time horizon hence the 4 year and 8-year timelines.

Page 8 line 166 in Revised manuscript with track changes

17. Line 136: The description for how indirect effects were calculated is a little confusing as written. Does infections averted by direct protection refer to the quantity calculated for direct effects?

**Response:** Yes, direct protection refers to the quantity calculated for direct PrEP effects.

Pages 8 to 9 lines 174 to 175 in Revised manuscript with track changes

18. Line 137: I believe this is the first time that the reference scenario (no PrEP) has been mentioned. It would be helpful to mention this earlier and describe how the reference scenario is used.

**Response:** We have clarified this point in a sentence added on line 147 (file with track changes) for the no PrEP scenario.

19. Line 139: For person-years required to prevent one infection, is this total effects? Only direct effects?

**Response:** This is total effects. To clarify this point, added the phrase, “regardless of whether the mechanism of protection is direct or indirect” on page 9 line 176.

Results:

20. Figures: Are there figure legends? I also noticed in Figure 3 that the scale on the y-axis is not the same across all plots, so the figure is misleading if the reader doesn’t look closely. The image quality is poor, so it was difficult to view the figures; this may be on the way the PDF is generated by Plos One, but make sure the image quality is sufficient.

**Response:** We have revised the graphs. Thank you for bringing this to our attention.

21. Line 145: By changing the age range that is targeted, does this imply more individuals are using PrEP? I.e., if it was 45% in men 15-24 and then 45% in men 15-49 (and similarly for women). Or is it the same number using PrEP but spread across a wider age range?

**Response:** It is 45% in men or women of the age group being targeted. We clarified this point in addressing suggestion #15 above.

22. Line 159: Are these 95% simulation intervals? It would be helpful to have a median value here since the interval seems wide and ratios less than 1 have a different interpretation than ratios above 1.

**Response:** These are not 95% simulation intervals. Indirect-to-direct ratios continue to increase over time hence the values given are for the beginning of the trajectory to the end of the trajectory of the 20-year period under study. We have revised the manuscript to clarify this point.

Discussion

23. Line 206: “Good-fitting parameter sets” is jargon that isn’t previously defined. If this is relevant for the Discussion, I suggest using plain language so readers without modeling experience can understand what this means.

**Response:** We have revised the manuscript to no longer use the jargon “good-fitting parameter sets.”

24. Line 212: This conclusion seems misleading, since PrEP was not targeted based on degree or concurrency specifically but allocated based on sex and age. These variables may correlate with degree and concurrency, but within groups PrEP was allocated equally.

**Response:** We agree that this is an overreach of what is supported by our analysis. We have removed the section that refers to concurrency.

**Reviewer #2:** This interesting manuscript addresses an important question – prioritization of offer of PrEP to different populations in western Kenya and South Africa in order to maximize impact in reducing new HIV infections. Overall, the manuscript is well written and provides important data to guide policy. I have several questions and suggestions for the authors to consider to strengthen the manuscript.

Major comments:
1) Because some national guidelines (e.g. in Kenya) now allow for offer of PrEP to persons at substantial risk of HIV acquisition, regardless of gender, it would be helpful to include an analysis in which PrEP is offered to both men and women at substantial risk. The current analyses in which PrEP is offered only to women or only to men are interesting exercises but are limited in terms of applicability to current programs. Although young women should understandably be prioritized for PrEP based on high incidence, men should also be prioritized for PrEP (as the authors note) to reduce incidence among women/their communities and, as many would argue, for their own health. Thus, the manuscript would be greatly strengthened by including an analysis in which PrEP is offered to both men and women at substantial risk.

**Response:** We have amended the Discussion section to be explicit about this important limitation of our analysis. As the Reviewer points out, programs will provide PrEP to a mix of population groups informed by the impact in each group as well as the accessibility, adherence, and demand for PrEP in each group. However, because HIV programs are still early in the process of PrEP scale-up, the proportions of age/sex groups represented in overall PrEP roll-out are not yet known. For this reason, we compared extreme scenarios (provision of PrEP to individuals of just a single age/sex group) to enable a clear comparison of the impact of PrEP in each group under different assumptions and over different time horizons.

Page 13 lines 268 to 273 in Revised manuscript with track changes

2) Could the authors please provide additional information on expected ART and VMMC coverage within the manuscript text and in Table 1 under different scenarios? E.g. when assuming status quo, do the guidelines referenced recommend ART treatment for all or based on CD4 thresholds, and what levels of ART coverage could that result in? How is ART coverage expected to differ by sex? This would help to contextualize the results.

**Response:** We have added references to indicate ART and VMMC coverage levels (Table 1 page 8 in Revised manuscript with track changes) for the status quo and fast -track scenarios. Additionally, we have added a table under Supplemental Appendix S2 to provide the number of people on ART by gender.

3) In the methods would be helpful to define “PrEP coverage” – is the denominator persons at substantial risk? All HIV-noninfected persons? If the latter, would be helpful to clarify this before Discussion.

**Response:** PrEP coverage is 45% of all HIV-negative individuals among men or women of a specific age group. We have clarified this in the manuscript in line 137 in Revised manuscript with track changes.

4) Community-level and population-level impact seem to be used at different times – could the authors kindly define these terms and if they are intended to be the same or different?

**Response:** We have changed all instances of population level to community-level. These terms were used interchangeably, and we thank the reviewer for encouraging us to use consistent terminology.

5) Given that the sexual network parameters impact the results significantly, it would be helpful to provide more information on network assumptions in the methods section

**Response:** We have re-written the methods to provide more detail regarding the sexual network component of the model, including references to more detailed analyses of the sexual network structure and its implications for HIV prevention.

Pages 4 to 5 lines 84 to 115 in Revised manuscript with track changes
Minor comments:
1) Could the authors also please provide more background on HIV prevalence and incidence in western Kenya and South Africa for readers with less knowledge of these regions?

**Response:** A summary of HIV epidemic in Kenya and South Africa has been added in the Supplementary Appendix overview page. The information is as follows:

**HIV epidemic in Kenya**

Approximately 1.6 million people were living with HIV in 2018 making Kenya's HIV epidemic the third-largest worldwide [1]. The HIV epidemic in Kenya is driven by sexual transmission and affects most of its general population though the most vulnerable groups are men who have sex with men, women, sex workers, young people (aged 15-24 years) and people who inject drugs [2]. In 2018, HIV incidence per 1000 uninfected among all people of all ages was 1.02 (CI: 0.6-1.75) and HIV prevalence among adults (15-49) was 4.7% (4%-5.7%) [1]. Approximately 89% of people living with HIV are aware of their HIV status, of which 82% (74% of all people living with HIV) are on HIV treatment and 92% (68% of all people living with HIV) are virally suppressed [2]. The most affected counties are located in the western part of Kenya which had 65% of all new HIV infections in 2018.HIV prevalence and ART coverage data for western Kenya counties considered in this study are given in this Supplementary Appendix S1 Table, S2 Table and S3 Table. Kenya approved the use of Pre-exposure prophylaxis (PrEP) in 2016 [2].

**References**

1. UNAIDS. UNAIDS Data 2019. <https://www.unaids.org/sites/default/files/media_asset/2019-UNAIDS-data_en.pdf>
2. Avert. HIV and AIDS in Kenya 2019. <https://www.avert.org/professionals/hiv-around-world/sub-saharan-africa/kenya>

HIV epidemic in South Africa

South Africa has the largest HIV epidemic with approximately 7.7 million people living with HIV in 2018 [1]. The HIV epidemic in South Africa affects all sections of the population including children, young people, adults, women and men [2]. Groups most affected by HIV in South Africa are women, young women and adolescent girls, sex workers, men who have sex with men, transgender women, children and orphans and people who inject drugs. In 2018, HIV incidence per 1000 population was 4.94 (CI: 4.43 - 5.51) and HIV prevalence among adults (15-49) was 20.4% (CI: 17.4% -22.5%) [1]. Approximately 90% of people living with HIV are aware of their HIV status, 62% of people living with HIV are on HIV treatment and 54% of people living with HIV are virally suppressed [1]. HIV prevalence and ART coverage data for South Africa used in this study are given in this Supplementary Appendix S1 Table and S2 Table South Africa approved the use of Pre-exposure prophylaxis (PrEP) in December 2015 [2].

References

1. UNAIDS. UNAIDS Data 2019. <https://www.unaids.org/sites/default/files/media_asset/2019-UNAIDS-data_en.pdf>
2. Avert. HIV and AIDS in South Africa 2019. <https://www.avert.org/professionals/hiv-around-world/sub-saharan-africa/south-africa>

2) Introduction notes that PrEP is a relatively high-cost intervention but discussion notes that costing data are limited – could the authors clarify what data are cited to indicate high cost of PrEP (noting that this may vary based on drug costs, personnel, etc)?

**Response:** Primary cost data for exact cost of PrEP delivery especially for countries in the sub-Saharan region is limited. The relatively high costs are derived from the few studies available hence we hope that by getting more data from current demonstration projects better cost-effectiveness analysis can be obtained as noted in the Discussion section on line 284 to 286 on page 13 Pages 4 to 5 lines 84 to 115 in Revised manuscript with track changes.

3) Methods – based on recent data (e.g. from AIDS2020) many individuals cycle on and off PrEP frequently. Could the authors comment on the impact of cycling back on PrEP in a shorter time than 6 months?

**Response: In** our previous analyses regarding the PrEP “cascade of prevention” (see Bershteyn et al., JIAS 2020 reference number 21 on page 18 in Revised manuscript with track changes) continuous use versus cycling on and off PrEP were equivalent for the same number of days covered by PrEP, provided those days had equivalent risk behavior. We now cite our previous analysis. We note in the Discussion section that the 6-month duration is a key simplifying assumption warranting further investigation (see lines 2640 to 266 – file with track changes).
