# Supplementary material for: Individual and community-level benefits of PrEP in western Kenya and South Africa: Implications for population prioritization of PrEP provision
Source: PLoS One. 2020 Dec 31;15(12):e0244761. doi: 10.1371/journal.pone.0244761 (PMC7775042; doi:10.1371/journal.pone.0244761)
Supplement: Attachment — Submitted filename: Response to Reviewers.docx [file pone.0244761.s004.docx]

**Response to Reviewers' comments:**

6. Review Comments to the Author

**Reviewer #1:** The authors have greatly improved the overall readability of the manuscript and clarity of the methods. My major concerns have been addressed and I only have a few minor comments at this stage. In addition, the manuscript would benefit from a careful edit for spelling and typos, as there are some minor mistakes throughout and I don’t think Plos One provides copy editing.

**Response:** Minor mistakes of spellings and typos have been edited.

1. The authors provided additional clarity about how PrEP effectiveness is operationalized in their model (i.e., 75% on average), which I appreciate. However, it remains unresolved that effectiveness is assumed to be equal between men and women, and between age groups. This is an important limitation, given that the primary study question is evaluating the population impact of allocating PrEP to different groups. For example, if adherence is lower among men (and thus the average effectiveness of PrEP is lower) then allocating PrEP to men may be far less effective on a population level, including indirect effects in later years, than projected. The authors need to spend more time discussing this limitation.

**Response:** We have added the following in the discussion section.

PrEP efficacy was assumed to be equal between men and women, and across age groups. This assumption has a direct effect on the community-level impact of allocating PrEP to different groups. For example, allocating PrEP to a group with low adherence would be less effective at a community-level, including indirect effects in later years.

2. Minor comment: Line 175 – I may have missed it, but it is still unclear what this interval represents. I appreciate the explanation in the response to my original question, but that’s not how I read it in the manuscript. On line 174, it says the ratios increase overtime, but it’s not clearly explained that the first number in the interval represents the beginning of the time horizon and the second number represents the end of the time horizon.

**Response:** We have added the following

The first number in the given indirect-to-direct ratio intervals represent the beginning of the time horizon (2016) and the second number represent the end of the time horizon (2036).

3. Minor comment: Line 223 – this says 250 simulations, but line 100 in the methods indicates 300 simulations.

**Response:** Thank you for picking this up. We have edited 300 to 250 simulations.

**Reviewer #2:** (No Response)
